# The Quantitative Evaluation of the Cell Structure Uniformity of Microcellular TPU with Low Porosity via a Digital Image Processing Method

**DOI:** 10.3390/ma17215203

**Published:** 2024-10-25

**Authors:** Liang Wang, Junjie Jiang, Wentao Zhai

**Affiliations:** 1 School of Materials Science and Engineering, Sun Yat-sen University, Guangzhou 510275, China; wangliang27@mail2.sysu.edu.cn (L.W.); jiangjj37@mail.sysu.edu.cn (J.J.); 2 Nanchang Research Institute, Sun Yat-sen University, Nanchang 330224, China

**Keywords:** digital image processing, microcellular TPU, cell structure uniformity, microcellular foaming, quantitative evaluation

## Abstract

The cell structure uniformity of microcellular polymers significantly impacts material performance, especially for low-porosity microcellular TPU used in chip polishing. The distribution of the cell structure of polishing pads directly affects the removal rate and process repeatability. Despite its importance, no quantitative method for evaluating cell structure uniformity has been reported in the literature. In this study, a digital image processing method that involves morphological operations of scanning electron microscopy (SEM) images, binarization, and cell localization, and the statistical evaluation of cell structure parameters was established to evaluate cell structure uniformity. A quantitative metric, the cell structure uniformity index (CUI), was calculated based on cell structure indices, incorporating the cell size index (*U*_d_), the cell number index (*U*_n_), and the cell local spacing index (*U*_r_). By establishing an ideal model and analyzing representative SEM images, the effectiveness and efficiency of the method for evaluating cell structure uniformity of microcellular TPU were successfully validated. The results demonstrated that low-porosity TPU foams exhibited relatively low cell structure uniformity compared to the ideal model. The heterogeneous nucleation process in TPU caused non-uniform cell structures due to the temporal and spatial non-homogeneities during the early cell nucleation process. As the cells grew, they merged and reduced the distance between them, resulting in improved cell structure uniformity.

## 1. Introduction

Microcellular foaming using carbon dioxide (CO_2_) or nitrogen (N_2_) is an advanced and environmentally friendly foaming technology for producing microcellular polymers with uniform cell structures [1,2,3]. Unlike conventional polymer foams, microcellular polymer foams generally exhibit micron-sized and even submicron-sized cell structures and are primarily fabricated using physical foaming agents rather than chemical blowing agents [4,5]. The tiny cell sizes endow microcellular foams with a lightweight nature and other desirable properties, such as enhanced impact resistance, high resilience, thermal and sound insulation, and a low dielectric coefficient. Microcellular polymers derived from thermoplastic elastomers, such as thermoplastic polyurethane (TPU), have been extensively employed in the fields of sports protection, thermal insulation, electronic packaging, and the chemical mechanical polishing (CMP) of semiconductor chips [6,7,8,9,10,11]. As one of the seven key processes in chip manufacturing, CMP involves the atomic-level removal of materials such as silicon, metal wires, and oxides to achieve both local and global surface planarization [12,13]. The core materials used in CMP include polishing slurries and polishing pads, and the CMP process combines chemical corrosion and mechanical friction. Polyurethane (PU) foams, PU/nonwoven fabric foams, and microcellular TPU serve as primary materials for top polishing pads, where their grooved structures facilitate the transport of polishing slurries and the removal of polished materials. The polishing properties of CMP pads depend on both intrinsic and extrinsic factors related to the polymer type and cell morphology [13].

**Figure 1:**
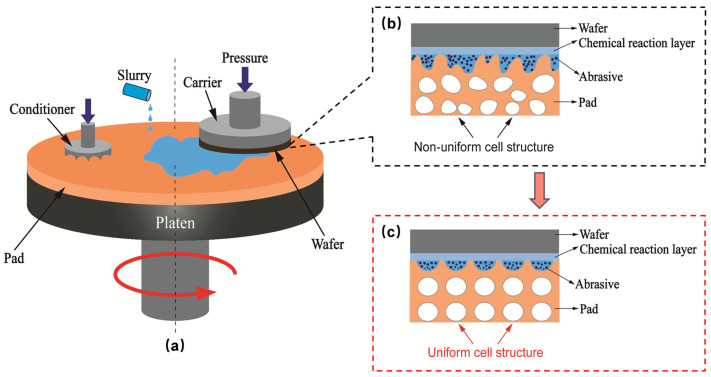
Schematic diagram of CMP process: (**a**) CMP equipment, (**b**) non-uniform cell structure, and (**c**) uniform cell structure for polishing top pad material.

The top pads contain numerous cell structures that serve various functions, including storing and homogenizing polishing slurry, securing nanoscale abrasives, and providing a micro-contact environment among the workpiece, polishing slurry, and polishing pad. To ensure that the polishing pads have appropriate compressibility and mechanical strength, microcellular TPU with low porosity, typically ranging from 10% to 50%, is generally selected for the top pads [14,15]. As illustrated in Figure 1, cell structure uniformity in the top pads significantly affects the distribution of the micro-contact environment. Uniform cell size and cell structure distribution can enhance polishing efficiency and improve stability and repeatability during the polishing process [16,17]. Therefore, developing low-porosity TPU foams with uniform cell structures is crucial for advancing high-performance microcellular TPU top pads. Since the advent of TPU microcellular foams, extensive studies have been conducted on foaming behavior [18,19], cell structure control [20,21,22] and structure–property relationships [23,24,25]. Although the properties and applications of polymeric foams are highly dependent on cell distribution, limited research has focused on quantifying cell structure distribution in low-porosity polymeric foams.

Digital image processing technology has been widely employed for the quantitative evaluation of particle distribution uniformity in composites, primarily by utilizing point patterns to describe the distribution characteristics of target particles [26,27,28]. These methods include regional statistics, nearest neighbor distance [27], and coordinate projection [28]. Kama et al. [29] used the regional statistics method, dividing the study area into multiple grids and calculating the number of particles within each grid. The uniformity of metal nanoparticles in the composites was quantified through statistical characteristics of particle numbers, such as the coefficient of variation (CV) or chi-squared distribution. Although this method is simple and straightforward, it does not account for the spatial distribution of particles. Tan et al. [30] utilized image processing techniques to quantify the uniformity of particles in noise reduction materials and established quantitative and local distance metrics to evaluate uniformity. The experimental results indicated that the statistical analysis was consistent with human visual observation, revealing that lower porosity corresponded to better uniformity. However, their method predominantly focused on the cumulative distribution function derived from nearest neighbor distances, limiting its ability to evaluate overall uniformity comprehensively. Pan et al. [31] proposed the mean crowding index, fractal dimension, and the CV of Voronoi cell areas to evaluate the abrasive distribution uniformity quantitatively from different perspectives. Nevertheless, treating particles as dimensionless points is inappropriate, which means that particle size should also be considered. Zhang et al. [32] reviewed advancements in quantitative evaluation methods for the arrangement of inclusions in composites, particularly highlighting the limitations of point patterns in uniformity assessment. They emphasized that, due to varying scales, particles could not simply be regarded as dimensionless points in both scientific research and practical applications, necessitating the consideration of factors such as size and shape, as exemplified in the analysis of concrete faults [33].

The cell structures of low-porosity polymer foams are usually formed at the early growth stage of nucleated bubbles. In a homogeneous nucleation system, cell nucleation occurs randomly within polymers, whereas in a heterogeneous nucleation system, there is temporal sequentiality and spatial selectivity [34]. This leads to diverse cell structures, including interconnected, merged, or closely connected cells, as well as varying cell sizes and shapes, such as circular and elliptical cells, ultimately causing non-uniformity in cell size and distribution. Traditional studies have often used qualitative methods to evaluate cell structure uniformity [35,36]. Frequently, foams have well-defined cell structure distribution, yet their cell location distributions may differ significantly, and this distinction cannot be explained by qualitative assessment [32]. Therefore, when evaluating the cell structure uniformity of polymeric foams, factors such as cell size, cell number, and cell local spacing should be considered in comprehensive quantitative analysis.

This paper proposed a method to quantify cell structure uniformity in SEM images of microcellular polymer foams. The method processed and analyzed SEM images, providing quantitative data on cell size, cell number, and cell local spacing, along with a corresponding graphical analysis. First, a quantitative approach for evaluating cell structure uniformity using digital image processing technology was proposed. Next, a set of evaluation indices for cell structure uniformity were established, introducing the cell structure uniformity index (CUI). The weights of three factors in the CUI, including the cell size index (*U*_d_), the cell number index (*U*_n_), and the cell local spacing index (*U*_r_), were determined. Additionally, through case studies, the method was applied to various cell structure scenarios to quantitatively evaluate cell structure uniformity. Finally, TPU foams with various cell structures and porosities were prepared, and the effects of foaming parameters on the uniformity of cell structures in TPU foams were investigated.

## 2. Methodology

### 2.1. Calculation of Actual Porosity

**Figure 2:**
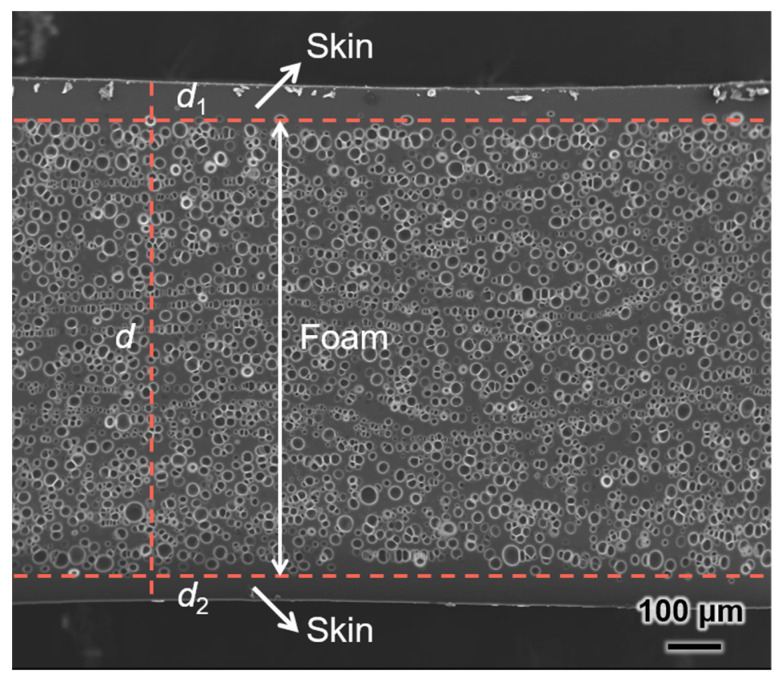
The cell morphology of microcellular TPU foam across the thickness direction.

During the microcellular foaming of polymers, the prepared samples often exhibit a non-foamed skin layer due to the rapid escape of dissolved gases from the polymer surface, which affects the actual foam porosity [37,38]. Therefore, it is necessary to calculate the actual foam layer density based on the skin layer thickness to correct porosity. To accurately determine skin layer thickness, the minimum distance from the skin layer to the nearest cell was first identified, followed by measuring the maximum distance from the skin layer to the majority of cells. The average of these two values was used to represent the skin layer thickness [39]. Figure 2 shows an SEM image of TPU foam fabricated based on a TPU resin with a shore hardness of 70D. The foam morphology is characterized by a skin layer on the top and bottom surfaces of the sample, with a porous structure in the middle. The foam layer density (*ρ*_2_) and actual porosity (*φ*) can be calculated using the following equations:

(1) $$\rho_{2}=\frac{\rho_{1}\;d-\rho_{0}\left(d_{1}+d_{2}\right)}{d-\left(d_{1}+d_{2}\right)}$$

(2) $$\varphi \;=1-\frac{\rho_{2}}{\rho_{0}}$$

where *d*, *d*_1_, and *d*_2_ represent the total thickness of the foamed sample, the top skin layer thicknesses, and the bottom skin layer thicknesses, respectively. *ρ*_0_ and *ρ*_1_ represent the sample density before and after foaming, respectively.

### 2.2. Processing Methods

**Figure 3:**
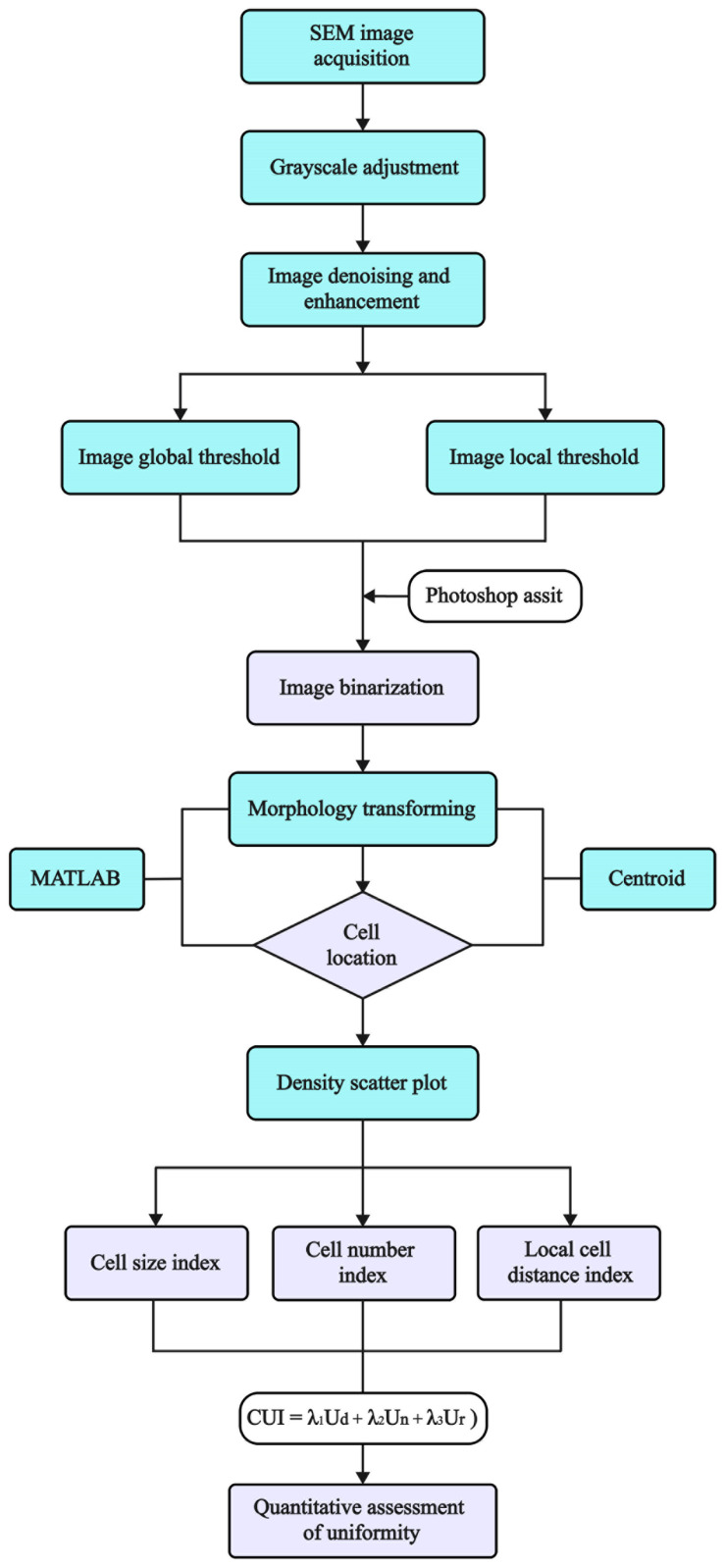
An overview of the digital image processing method for quantitatively evaluating cell structure uniformity.

Digital image processing technology uses computers to process digital images, extract useful information, and perform various operations to achieve specific objectives. In this paper, an analysis program for obtaining quantitative parameters of cell structure uniformity was developed based on MATLAB’s programming and image-processing functions [40,41], along with relevant statistical principles [42]. The SEM images were successfully digitized by running this program in MATLAB. As shown in Figure 3, the program involved four main steps: morphological processing, binarization, cell localization, and the statistical quantification of uniformity indexes. This approach quantitatively assessed the cell structure uniformity through three indices: *U*_d_, *U*_n_, and *U*_r_. Additionally, the CUI was introduced to represent the degree of uniformity. The program was applicable to almost all types of polymeric foams.

### 2.3. Processing Steps

#### 2.3.1. Image Binarization

Binarization is the process of converting a color or grayscale image into a black-and-white image. During this process, SEM images with appropriate contrast and magnification are selected and processed in MATLAB for morphological adjustments, such as grayscale correction, denoising, and inversion, to enhance image quality. Image binarization can be mainly divided into global threshold and local threshold methods. The global threshold method only considers grayscale values but ignores the spatial characteristics of an image, so it is highly sensitive to noise. The local threshold method calculates a threshold based on this information in the field of each pixel on the image, and then selects different thresholds for segmentation in different image areas, which only considers local features of an image but ignores the overall distribution. Therefore, to obtain a binary image with relatively large differentiation between cells and background, this paper proposed a local iteration Otsu algorithm, combining the global threshold and local iteration threshold methods.

To facilitate the subsequent morphological processing and cell localization of SEM images, the improved Otsu method was employed to determine the final segmentation threshold, while histogram equalization was applied to enhance the grayscale and contrast of SEM images [30]. However, some binarized images may exhibit defects such as partially missing cells, merged cells, spurs, and noise, which can affect the quality of binarization. For images with poor results, Photoshop was used for further refinement to better distinguish cells from the background while preserving the original cell features as much as possible. In the binarized images, cell regions appear in black, while the background is displayed in white.

**Figure 4:**
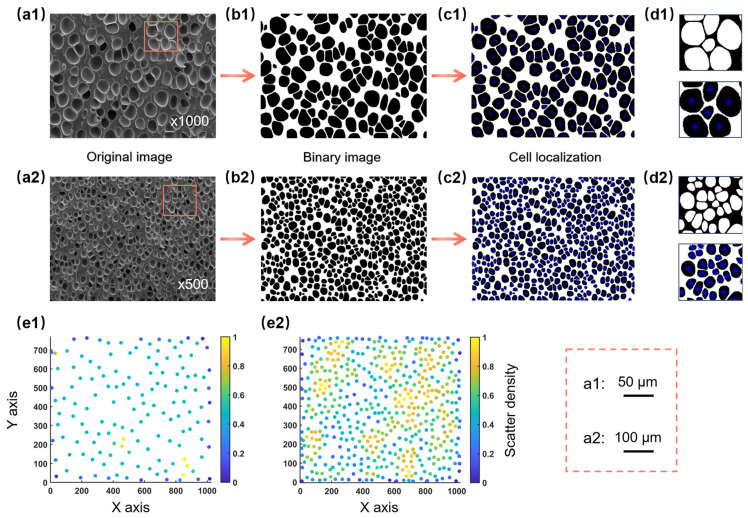
An illustration of the different stages of image processing: (**a1**,**a2**) original images, (**b1**,**b2**) binary images, (**c1**,**c2**) cell localization, (**d1**,**d2**) negative images, and (**e1**,**e2**) density scatter plots.

Figure 4 illustrates the binarization and cell localization process for SEM images of the same foamed sample at different magnifications. Figure 4(a1,a2) shows the original SEM image of the foamed sample, where the red region corresponds to the magnified images shown in Figure 4(d1,d2), illustrating the effective results of the binarization process. and Figure 4(b1,b2) presents the binarized image. Figure 4(c1,c2) is a schematic of cell localization, achieved by calculating the centroid position of cells (marked with blue asterisks). Figure 4(d1,d2) shows the local magnification of inversion and cell localization, displaying the precise positioning of each cell in the local area. The results demonstrate that this method achieved effective binarization and successfully extracted cell structure information from SEM images.

#### 2.3.2. Density Scatter Plot

A density scatter plot is an enhanced scatter plot that provides intuitive data visualization by reflecting the distribution of points and local density variations through color mapping. Seo et al. [43] applied the scatter plot to assess particle distribution uniformity and identify anomalous regions in composites. This paper innovatively introduced density scatter plots to visually analyze the number and distribution characteristics of cells in SEM images, achieving a qualitative evaluation of overall cell structure uniformity.

In practice, a coordinate matrix was first created in MATLAB, converting the image coordinate system into a mathematical one, and the coordinates of each centroid point relative to the bottom of the image were calculated. The scatplot function was then used to plot density scatter plots of the SEM images, where each cell was represented by its centroid in the coordinate system. To evaluate the uniformity of cell distribution, the local outlier factor (LOF) was introduced as a density-based outlier detection method. The LOF evaluated the degree of outliers by comparing the local density of each data point with that of its neighboring points, calculating the local reachable density (LRD) of each point, and normalizing it within the range of 0 to 1. A gradient scheme from blue to yellow was used to reflect the density variations, with the corresponding color determined by the normalized LRD value of each point. Figure 4(e1,e2) displays the density scatter plots at different magnifications, with the right column showing the degree of point aggregation to evaluate cell clustering. Compared to Figure 4(e1), Figure 4(e2) contains more points in total, with a greater number of yellow points that were more dispersed, indicating closer cell packing and smaller cell spacing. Thus, sample (a2) exhibited higher cell structure uniformity, which aligned with human visual observations [26,29].

#### 2.3.3. Establishment of Quantitative Uniformity Indices

To quantitatively characterize cell structure uniformity, this study utilized the MATLAB (R2022b) and ImageJ (1.53v 21 November 2022) software to statistically analyze relevant parameters of cell structures. Three quantitative metrics for cell structure uniformity were established, as detailed in Table 1. Uniformity was represented by the CUI, where a lower CUI value indicated a higher degree of uniformity. Through these indices, cell structure uniformity was systematically quantified.

**Figure 5:**
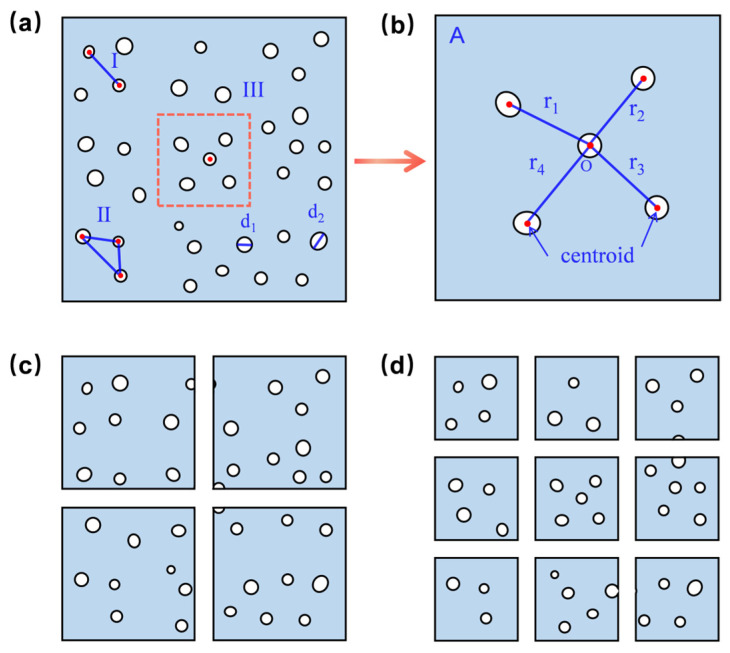
The process schematic of obtaining quantitative indexes of cell structure uniformity: (**a**) overall schematic, (**b**) schematic diagram for calculating local spacing of cells, and (**c**,**d**) schematic diagrams of four and nine equal parts, respectively.

This study proposed a statistical method based on standard deviation (*S*) and the coefficient of variation (*CV*) to measure data dispersion. The *CV*, defined as the ratio of the standard deviation to the mean, was used to compare variability between two or more data groups with different means. A smaller *CV* value indicated higher data stability. To evaluate the distribution behavior of cells in different images, the *CV* was introduced as a dimensionless quantity to reflect dispersion and uniformity. The greater the number of cells, the more accurate the *CV* measurement. Figure 5 illustrates the method for obtaining the quantitative uniformity indices. In Figure 5a, I and II demonstrate the calculation of the distance between cells. Figure 5b illustrates the calculation of the average cell distance within region A, which is a magnified view of region III. Using MATLAB’s image process toolbox, a binarized image was divided into subregions of equal area, where the number of cells, cell size, and cell local spacing were calculated for each subregion. Subsequently, their standard deviations and coefficients of variation were computed. By integrating the indices of *U*_d_, *U*_n_, and *U*_r_, the CUI value was calculated eventually. The higher the CUI value, the lower the cell structure uniformity. Notably, cell spacing was calculated by converting the pixel value into the actual scale of the SEM images. The formulas for calculating these indexes are as follows:

(3) $$S\;=\sqrt{\frac{\sum_{i=1}^{N}{\left(x_{i}-u\right)}^{2}}{N-1}}$$

(4) $$\mathrm{CV}\;=\frac{S}{\overset{-}{N}}$$

(5) $$\mathrm{CUI}=\sum_{i=1}^{3}\lambda_{i}U,\;\lambda_{1}+\lambda_{2}+\lambda_{3}=1$$

where *N* is the total number of data points, $\overset{-}{N}$ is the mean, *S* is the standard deviation, and *CV* is the coefficient of variation. *U* denotes the quantification index of uniformity, *CUI* represents the cell structure uniformity index, and *λ*_i_ is the weight of each index. Since the influence of each index on uniformity is considered equal, *λ*_i_ is set to 1/3, meaning all indices have the same weight.

Cell size is a crucial parameter for characterizing cell structure; thus, the cell size index (*U*_d_) was introduced to assess the uniformity of cell size. The cell sizes of polymeric foams typically range from hundreds of nanometers to hundreds of micrometers. Existing studies often used the standard deviation (*S*) of cell size to describe its distribution, but this method becomes less effective when there are large differences in cell size [44]. The standard deviation can be influenced by the mean, particularly when there are significant discrepancies in average values or inconsistent units, making direct comparisons potentially inaccurate. In contrast, the coefficient of variation (*CV*) better avoids such issues. In Figure 5a, the average cell size and the *CV* of cell size within each region were calculated using ImageJ. The average of each *CV* (denoted as *CV*_1_) was calculated as the *U*_d_ to evaluate cell size uniformity. A smaller *U*_d_ value indicated less variation in cell size across subregions, suggesting that the overall distribution was more uniform. Additionally, a histogram of cell size distribution was plotted to achieve a quantitative evaluation of both overall and local cell size uniformity.

Cell density is another important parameter for characterizing cell structure; thus, the cell number index (*U*_n_) was introduced to assess the uniformity of cell distribution. The equal-area partitioning method, as shown in Figure 5c,d, divided the image into four and nine sub-regions and counted the number of cell centroids in each subregion using the count function. Similarly, the average *CV* of the cell number (denoted as *CV*_2_) was then calculated as *U*_n_ for a simple evaluation of cell distribution uniformity. A smaller *CV*_2_ value indicated a more uniform local cell distribution, while a smaller *U*_n_ value overall meant less variation in cell number distribution in subregions, suggesting a more uniform overall cell distribution. It was worth noting that to avoid boundary issues that may affect the uniformity calculation, cells were assigned according to their centroid positions. If the center of the centroid was located within a subregion, the cell was counted in that region, ensuring the reliability of the results.

The previously discussed indices based on cell size and cell number do not account for spatial distribution information. When cells are concentrated in certain areas of SEM images, the uniformity quantification model may yield inaccurate results. Therefore, the introduction of a local cell spacing index (*U*_r_) was necessary. The calculation method for *U*_r_ is shown in Figure 5b. Suppose there were five cells in subregion A. For any given cell with centroid O, the distances between O and the centroids of the four surrounding cells were calculated using the pdist2 function in MATLAB. The average of these distances, denoted as $\bar{r_{1}}$, was then computed. Similarly, the average distances $\bar{r_{2}}$ to $\bar{r_{4}}$ for the remaining cells were calculated. The overall average R_A_, representing the local cell spacing for subregion A, was obtained by averaging $\bar{r_{1}}$ to $\bar{r_{4}}$. Other subregions were processed similarly. To assess the spatial distribution of cells, the coefficient of variation of cell spacing (denoted as *CV*_3_) and the mean of *CV*_3_ (denoted as *U*_r_) in each subregion were calculated. Smaller and closer *CV*_3_ values indicated less variation in cell spacing within subregions, reflecting better local cell distribution uniformity. A smaller *U*_r_ value suggested an overall better uniformity of cell distribution. These methods provided as effective means to quantitatively evaluate both global and local uniformity of cell structure distribution.

## 3. Results and Discussion

### 3.1. Cell Structure Evolution of Low-Porosity Microcellular Polymers

In the microcellular foaming of polymers, supercritical CO_2_ or N_2_ fluids diffuse into the polymer matrix under high temperature and pressure, gradually reaching a dissolution equilibrium with a solubility of 0.5–20.0% [3]. For the pressure quench foaming process, when the system is released to atmospheric pressure, the supercritical fluid becomes supersaturated within the polymer, leading to cell nucleation. Once the size of the nucleated bubbles exceeds the critical nucleation size (theoretically in the range of a few nanometers) [45], these bubbles grow spontaneously. The supercritical fluid continues to diffuse into the bubbles, promoting cell growth. As the foaming system cools, the modulus of the foamed material increases, molecular chain movements are frozen, and the cell structure stabilizes.

According to classical nucleation theory and extensive visualization studies, factors such as polymer/fluid interfacial tension, nucleating agents, and the stress field play important roles in cell nucleation [4,46,47]. During microcellular foaming, cell nucleation occurs unevenly across both temporal and spatial scales, resulting in a non-uniform distribution of nucleated bubbles [21]. However, as the cells grow, the cell structure merges and even coalesces [48], reducing the distance between cells and thinning the cell walls, ultimately leading to a more uniform cell structure.

Low-porosity TPU microcellular foams, with a typical porosity of 10–50%, are applied in wafer polishing. These materials feature widely spaced cells and thick cell walls, with cell structures in which nucleation is essentially completed, although growth is not yet fully completed. The selected cell structures below all exhibit characteristics of low porosity.

### 3.2. Applications of Uniformity Quantification Method

#### 3.2.1. Number of Analyzed Cells

**Figure 6:**
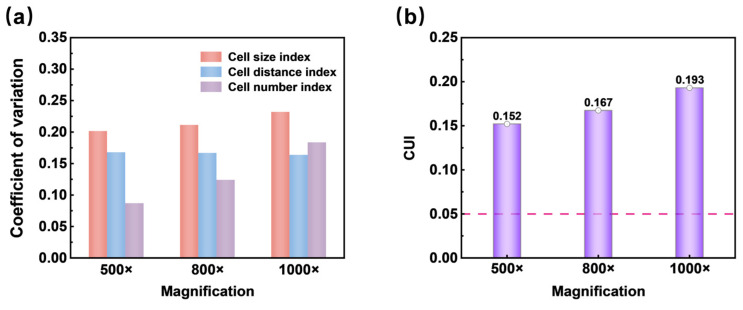
(**a**) The CV of three uniformity index and (**b**) CUI values corresponding to SEM images with different magnifications.

The developed program allows for measuring the number of cells in each SEM image. Cell recognition can be enhanced by using appropriate magnification during micrograph acquisition [49]. Therefore, the CUI values of images with different magnifications were analyzed for the same foam sample section (shown in Figure 4). Figure 6a demonstrated the various uniformity indexes corresponding to different magnifications. Figure 6b showed the CUI values corresponding to SEM images with different magnifications, where the red dashed line represented the CUI value of the ideal model. From the data in Figure 6b, it was evident that the CUI values varied with magnification. As magnification increased, the *U*_n_ value significantly increased and the *U*_d_ value gradually increased, while the *U*_r_ value remained essentially unchanged. Consequently, the CUI value increased from 0.152 to 0.193, indicating a decrease in cell structure uniformity with higher magnification, which aligned with visual observations. Specifically, for the SEM images at three magnifications (500×, 800×, and 1000×), the corresponding number of cells were 476, 190, and 139, respectively. In the image with a magnification of 500×, computational complexity increased due to the excessive number of cells. Meanwhile, visual observations suggested that the cell structure itself was relatively uniform. Thus, under these conditions, further quantification of uniformity became less meaningful. In this case, the SEM image with a magnification of 800× was ultimately selected for quantifying cell structure uniformity, as it yielded a smaller CUI value. Selecting an appropriate magnification before quantifying uniformity is crucial to ensure that the number of cells is around 150–200, making the quantification method more feasible and applicable.

#### 3.2.2. The Establishment of the Ideal Model

**Figure 7:**
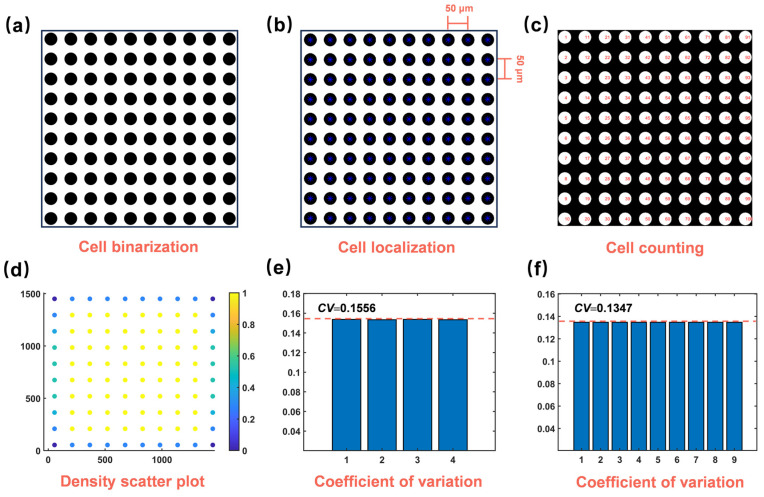
A schematic diagram of an ideal model with a completely uniform cell structure and its evaluation of uniformity: (**a**) cell binarization of ideal model, (**b**) cell localization of ideal model, (**c**) cell couting of ideal model, (**d**) density scatter plot of ideal model, (**e**) the cofficient of variation of cell distance of ideal model with four subdivisions, (**f**) the cofficient of variation of cell distance of ideal model with nine subdivisions.

The previous section introduced a quantitative method for evaluating cell structure uniformity but lacked a specific benchmark. Therefore, an ideal model of absolute uniformity was established, with its cell structure uniformity quantified as shown in Figure 7. This model contained 100 cells (i.e., 10 by 10) of the same size, with their positions distributed uniformly. Calculations showed that both *U*_n_ and *U*_d_ values for this model were 0, while the *U*_r_ value depended on the number of subdivisions. Figure 7e,f showed that *U*_r_ values for four and nine subdivisions were 0.156 and 0.135, respectively, with little difference. Considering the appropriate number of cells, the nine-division method for calculating the *CV* value was more suitable. According to Equation (5), the CUI value of the ideal model was calculated as 0.045, which was very small, indicating absolute uniformity. Additionally, the CUI value for ideal models with 121, 144, 169, and 196 cells was calculated as 0.049, 0.050, 0.051, and 0.052, respectively. Since the ideal model should have a completely uniform distribution of cell sizes and positions, the minimum CUI value (CUI_0_ = 0.050) was used as the reference benchmark for subsequent evaluations. In the following sections, typical SEM images with different cell structures will be evaluated for uniformity to verify the accuracy and reliability of the quantitative uniformity method. The selected images included varying cell sizes, porosities, and morphologies, with the number of cells approximately between 150 and 200.

#### 3.2.3. Uniformity Evaluation of SEM Images with Different Cell Sizes

**Figure 8:**
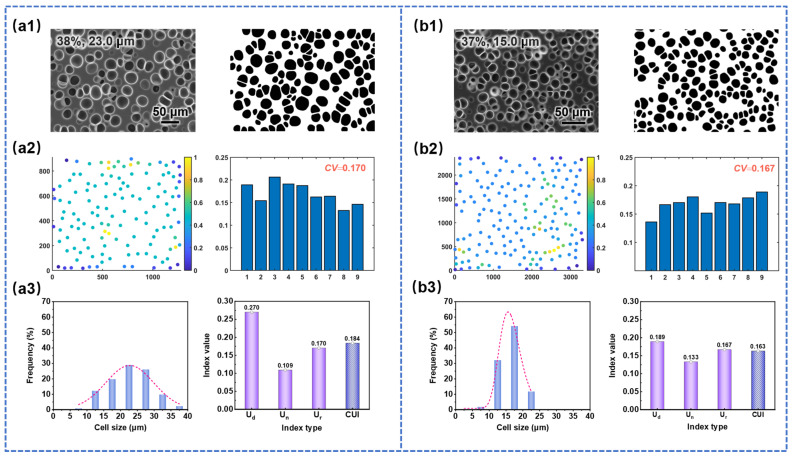
Quantitative evaluation of cell structure uniformity of SEM images with different cell sizes: (**a1**,**b1**) original SEM images and binary images, (**a2**,**b2**) density scatter plots and the CV of cell distance of SEM images with nine subdivisions, (**a3**,**b3**) cell size distribution histograms and the quantified uniformity indexes.

The selected SEM images with different cell sizes, along with their cell uniformity evaluation results using three indices, are presented in Figure 8(a3,b3). Specifically, Figure 8(a1,b1) showed binary images, Figure 8(a2,b2) presented density scatter plots and histograms of coefficient of variation for cell spacing (*U*_r_), and Figure 8(a3,b3) displayed cell size distribution histograms and the quantified uniformity indices (the same below). From Figure 8, it can be observed that the cell size distribution of sample (b1) was narrower compared to that of sample (a1), corresponding to the smaller *U*_d_ value of sample (b1). Moreover, the *U*_d_ value of sample (b1) was smaller than that of sample (a1), while the U_n_ value was larger and the *U*_r_ value was similar. This resulted in a smaller CUI value for sample (b1) (0.163 < 0.184), indicating that the cell structure uniformity of sample (b1) was superior to that of sample (a1). The experimental statistical results were consistent with the analysis reported in the literature and visual observations [26,50].

#### 3.2.4. Uniformity Evaluation of SEM Images with Different Porosities

**Figure 9:**
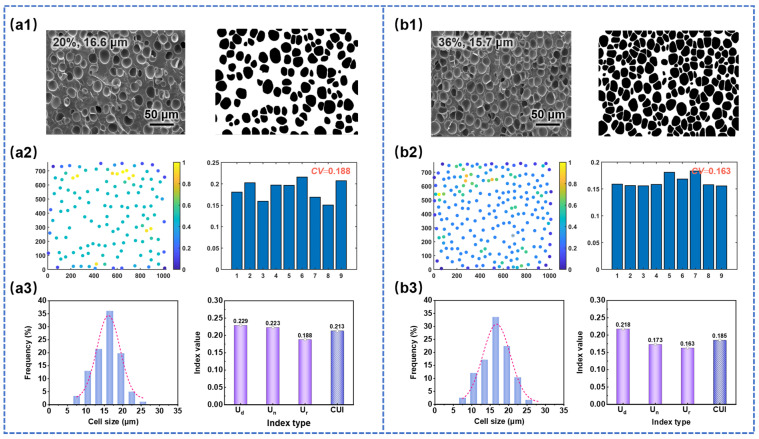
Quantitative evaluation of cell structure uniformity of SEM images with different porosities: (**a1**,**b1**) original SEM images and binary images, (**a2**,**b2**) density scatter plots and the CV of cell distance of SEM images with nine subdivisions, (**a3**,**b3**) cell size distribution histograms and the quantified uniformity indexes.

SEM images with different porosities were selected, and their uniformity quantification results are displayed in Figure 9. As shown in Figure 9(a3,b3), the cell size distributions of sample (a1) and sample (b1) were similar, corresponding to their comparable *U*_d_ values. It can be concluded that sample (b1) exhibited better cell structure uniformity, as the three index values for sample (b1) were smaller than those for sample (a1), resulting in a lower CUI value (0.185 < 0.213). The experimental statistical results aligned with the visual observation of the original SEM images [26].

#### 3.2.5. Uniformity Evaluation of SEM Images with Different Cell Morphologies

**Figure 10:**
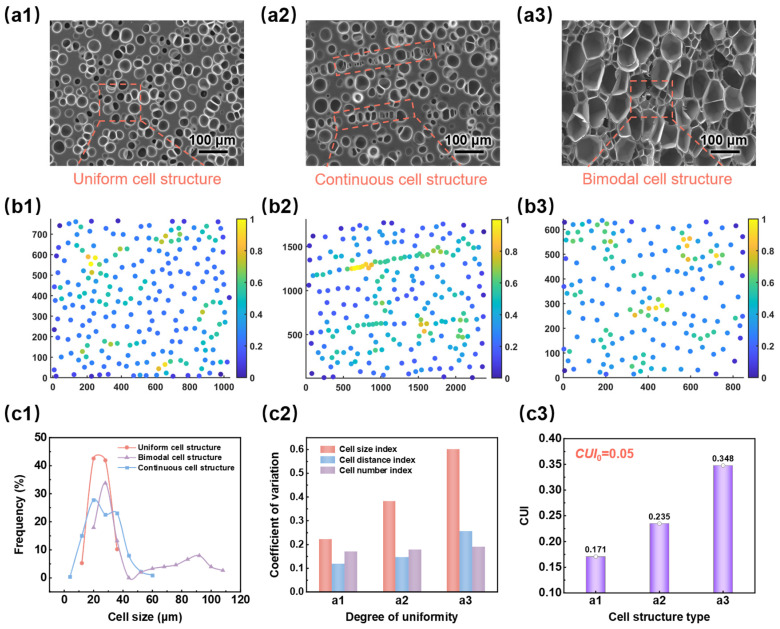
Quantitative evaluation of cell structure uniformity of SEM images with different cell morphologies: (**a1**–**a3**) original SEM images with different cell morphologies; (**b1**–**b3**) density scatter plots corresponding different cell morphologies; (**c1**) cell size distribution histograms corresponding different cell morphologies; (**c2**) the CV of three uniformity indexes of different cell morphologies; (**c3**) the CUI of SEM images with different cell morphologies.

SEM images of three different cell morphologies were selected, and their uniformity quantification results are illustrated in Figure 10(c1–c3). Specifically, Figure 10(a1–a3) displays the uniform structure, continuous structure, and bimodal cell structure, each containing approximately 200 cells. Figure 10(b1–b3) shows the corresponding density scatter plots, where yellow points accurately reflected the characteristics of each cell morphology. Figure 10(c1) shows the cell size distribution of foamed samples, and Figure 10(c2) presents histograms of uniformity quantification indices. It was observed that the cell size distribution for the three morphologies widened sequentially, leading to poorer size distribution uniformity, which aligned with the increasing cell size index in Figure 10(c2). Figure 10(c2,c3) shows that all three quantitative uniformity indices of samples (a1–a3) gradually increased, resulting in rising CUI values (0.171 < 0.235 < 0.348). This suggests that the cell structure uniformity of the three samples deteriorates sequentially, consistent with the visual observations.

#### 3.2.6. Classification of Cell Structure Uniformity

**Figure 11:**
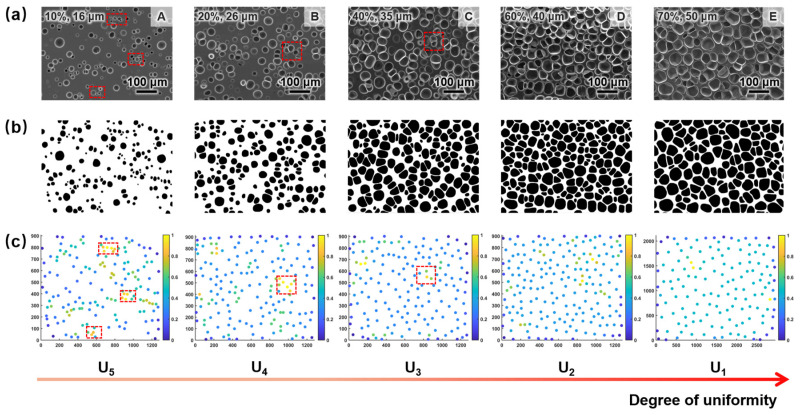
(**a**) SEM images, (**b**) binary images, and (**c**) density scatter plots describing the qualitative evaluation of various uniformity levels.

To standardize the evaluation of cell structure uniformity in SEM images for subsequent studies, it is essential to classify the uniformity levels [29]. In this study, SEM images of numerous cross-sections of TPU foam samples were collected for analysis. Their porosities were calculated to be between 10% and 70%, with average cell sizes ranging from 15 to 50 μm, according to Equation (2). Five uniformity levels were considered, as shown in Figure 11A–E: uneven uniformity, low uniformity, relatively low uniformity, medium uniformity, and high uniformity (denoted as U_5_ to U_1_). The cell structure uniformity improved gradually from U_5_ to U_1_. Observing from left to right, the size and positional distribution of cells became increasingly uniform as cell size and porosity increased. In the density scatter plots, the clustering of points and the number of yellow points both decreased and the distribution of scattered points tended to be uniform, which qualitatively indicated an improvement in cell structure uniformity.

**Figure 12:**
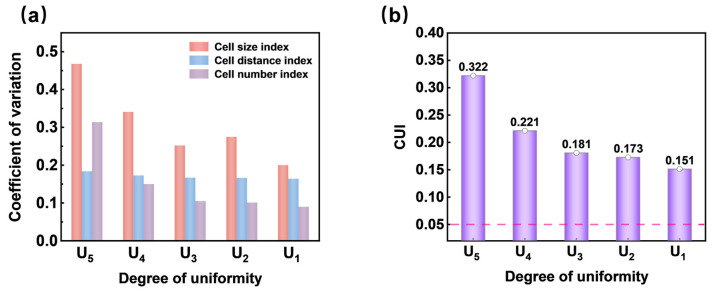
(**a**) The CV values of U_5_–U_1_ of three factors and (**b**) the CUI values corresponding to the cell structure of various uniformity levels.

Figure 12 presents the quantitative uniformity characterization results for SEM images A–E. As illustrated in Figure 12a, the three uniformity quantitative indices for these images exhibited a decreasing trend, leading to a gradual reduction in CUI values (0.322 > 0.221 > 0.181 > 0.173 > 0.151). This indicated an increase in cell structure uniformity. For instance, the cell structure represented by U_1_ showed high uniformity, with a CUI value of 0.151, which was close to the ideal model’s CUI_0_ value (0.050). Consequently, the CUI value can be used to determine the uniformity levels of cell structures depicted in the SEM images.

#### 3.2.7. Materials and Sample Preparation

TPU pellets, with a shore hardness of 70D, were purchased from Lubrizol Specialty Chemicals Manufacturing (Shanghai) Co., Ltd., CO_2_ with a purity of 99.9% was purchased from Guangzhou Guangqi Gas Corporation. TPU pellets were completely dried at 100 °C for 4 h and then hot-pressed into disks with a thickness of 1 mm. The preparation of TPU foams was based on our previous work [21]. Briefly, the TPU disks were placed inside the high-temperature chamber, and then the chamber was closed by a hydraulic ram. Then, CO_2_ was fed into the chamber by a high-pressure syringe pump. After being saturated for various saturation times, the supercritical fluid was rapidly released in 5 s intervals by a solenoid valve. The saturation pressure and saturation time were 10–15 MPa and 30 min, respectively. It is worth noting that the microcellular TPU foams, with a porosity of 10–50%, had a number of cells approximately between 150 and 200 in the selected SEM images.

#### 3.2.8. Evolution of Cell Structure Uniformity of Microcellular TPU Foams

**Figure 13:**
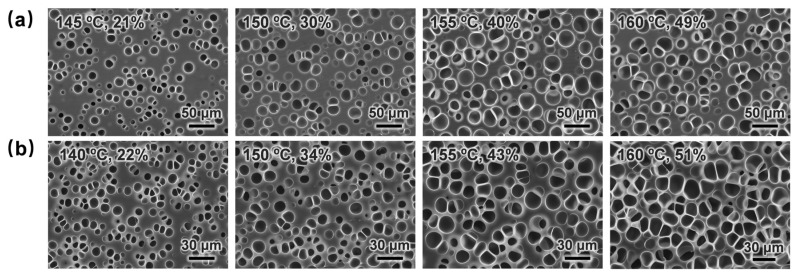
Cellular morphologies of TPU foams prepared at (**a**) 10 MPa and (**b**) 15 MPa and different foaming temperatures.

**Figure 14:**
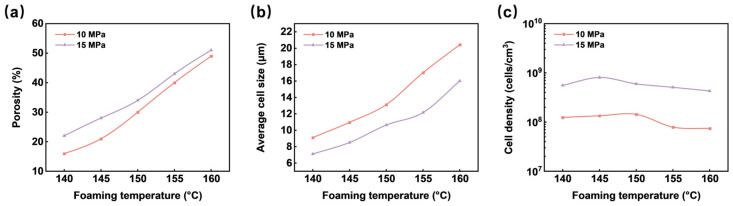
The evolution of porosity (**a**), average cell size (**b**), and cell density (**c**) of the TPU foams as a function of the foaming temperature.

The effects of foaming temperature and saturation pressure on the cell structure uniformity of TPU foams with low porosity were investigated. Figure 13 illustrates the SEM images of the foams, and Figure 14 provides data on the porosity, average cell size, and cell density of the foams. As the foaming temperature increased, the cell structure transitioned from circular to elliptical, accompanied by an increase in cell size and a decrease in cell spacing, leading to a rise in foam porosity. This phenomenon was attributed to the rise in foaming temperature, which reduced the modulus of the polymer matrix and enhanced the diffusion rate of the foaming agent, thereby facilitating cell growth and foam expansion. Figure 14c demonstrates that the cell density remained relatively constant across varying foaming temperatures but increased with higher saturation pressures. This was attributed to the greater solubility of the supercritical fluid, which reduced the energy barrier of cell nucleation, resulting in a higher number of nucleated cells.

**Figure 15:**
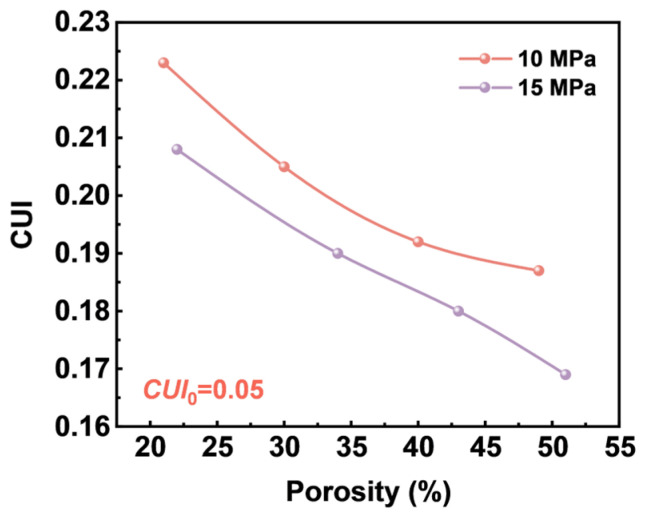
The CUI values with the porosity of TPU foams.

The relationship between the CUI values and the porosity of TPU foams is illustrated in Figure 15. It was observed that as porosity increased, the CUI values gradually decreased, indicating an enhancement in the uniformity of the cell structure. This phenomenon can primarily be attributed to spatial and temporal non-homogeneities during cell nucleation, which resulted in non-uniform cell structures. Additionally, an increase in foaming temperature led to higher porosity, promoting further cell growth, reducing the cell spacing between them, and thinning the cell walls, thereby enhancing cell structure uniformity. Furthermore, the results indicated that all foams prepared under a saturation pressure of 15 MPa displayed CUI values lower than those prepared at 10 MPa, suggesting that increased saturation pressure was beneficial for achieving more uniform cell structures. This improvement can be explained by the fact that higher saturation pressures facilitate increased gas solubility in the TPU matrix, significantly lowering the energy barrier of cell nucleation, increasing the number of nucleated cells, and consequently resulting in smaller cell sizes and enhanced cell density. As a result, the foams exhibited a more uniform cell structure. Overall, under heterogeneous nucleation, the cell structure uniformity of TPU foams was relatively uniform. However, there remained a certain discrepancy compared to the ideal model, particularly at lower porosities where the uniformity of cell structure was less uniform.

## 4. Conclusions

The cell structure uniformity of microcellular polymers significantly impacts material performance. This study proposed a new method for quantifying cell structure uniformity by considering cell size, cell number, and cell local spacing factors. The degree of cell structure uniformity of microcellular TPU was evaluated by comparing CUI values. The experimental results indicated that the statistical analysis was consistent with visual observations. Smaller CUI values indicated better uniformity. By constructing an ideal model, analyzing typical cases, and comparing it with other established methods, the effectiveness and efficiency of the method were validated. The results demonstrated that low-porosity TPU foams exhibit relatively low cell structure uniformity compared to the ideal model. The heterogeneous nucleation process in TPU led to non-uniform cell structures, arising from the temporal and spatial non-homogeneities during the initial cell nucleation. As the cells grew, they merged and reduced the distance between them, resulting in improved cell structure uniformity. It is expected that the CUI will be utilized by the research community as a tool for quantitatively exploring cell morphology in microcellular polymers.

## Figures and Tables

**Table 1:** Quantitative evaluation indexes of cell structure uniformity.

Uniformity Index	Evaluation Method	Evaluation Effect
Cell size index (*U*_d_)	*CV*_1_ histogram	Evaluation of the overall and local uniformity of cell size distribution
Cell number index (*U*_n_)	*CV*_2_ histogram	Simple evaluation of cell distribution uniformity
Cell spacing index (*U*_r_)	*CV*_3_ histogram	Precise evaluation of cell distribution uniformity

## Data Availability

The original contributions presented in the study are included in the article; further inquiries can be directed to the corresponding author.
